# Linking the morphological and metabolomic response of *Lactuca sativa* L exposed to emerging contaminants using GC × GC-MS and chemometric tools

**DOI:** 10.1038/s41598-017-06773-0

**Published:** 2017-07-26

**Authors:** Carlos Hurtado, Hadi Parastar, Víctor Matamoros, Benjamín Piña, Romà Tauler, Josep M. Bayona

**Affiliations:** 1 Department of Environmental Chemistry, IDAEA-CSIC, c/Jordi Girona, 18-26, E-08034 Barcelona, Spain; 2 Department of Chemistry, Sharif University of Technology, Tehran, Iran

## Abstract

The occurrence of contaminants of emerging concern (CECs) in irrigation waters (up to low μg L^−1^) and irrigated crops (ng g^−1^ in dry weight) has been reported, but the linkage between plant morphological changes and plant metabolomic response has not yet been addressed. In this study, a non-targeted metabolomic analysis was performed on lettuce (*Lactuca sativa* L) exposed to 11 CECs (pharmaceuticals, personal care products, anticorrosive agents and surfactants) by irrigation. The plants were watered with different CEC concentrations (0–50 µg L^−1^) for 34 days under controlled conditions and then harvested, extracted, derivatised and analysed by comprehensive two-dimensional gas chromatography coupled to a time-of-flight mass spectrometer (GC × GC-TOFMS). The resulting raw data were analysed using multivariate curve resolution (MCR) and partial least squares (PLS) methods. The metabolic response indicates that exposure to CECs at environmentally relevant concentrations (0.05 µg L^−1^) can cause significant metabolic alterations in plants (carbohydrate metabolism, the citric acid cycle, pentose phosphate pathway and glutathione pathway) linked to changes in morphological parameters (leaf height, stem width) and chlorophyll content.

## Introduction

Contaminants of emerging concern (CECs), including compounds such as pharmaceuticals and personal care products, are increasingly detected in agricultural irrigation waters as a consequence of multiple inputs throughout the water cycle and partial removal during water reclamation and potabilisation^1–3^. Both plant uptake of CECs and changes in plant morphological (e.g. biomass production and shoot growth) and physiological (e.g. phytohormones and chlorophyll content) parameters have been reported under different experimental conditions^4–8^. Plants can transform the uptaken CECs through plant detoxification mechanisms involving enzymatic (phase I) and conjugation (phase II) processes^4, 5^. For instance, the presence of CECs can increase antioxidant enzymatic activities due to detoxification processes^6^. Plants are known to be able to biosynthesise specialised or secondary metabolites to adapt to biotic or abiotic environmental stressors, such as drought, salt, low soil oxygen, metals, temperature, light and oxidative stress^7, 8^. Nevertheless, the application of metabolomics to study plant response to CEC exposure in agricultural irrigation waters has not yet been investigated.

Metabolomics is the comprehensive analysis of all of an organism’s metabolites to understand the complexity of molecular interactions in biological systems^9, 10^. Plant metabolomics aims to study the plant system at the molecular level to provide a non-biased characterisation of the total metabolite pool (metabolome) of a plant’s tissue in response to its environment^7^. Plant metabolomics includes the analysis of a wide range of chemical compounds, from ionic inorganic compounds to biochemically derived hydrophilic carbohydrates, organic and amino acids, and a range of hydrophobic lipid-related compounds. Analytical methodologies based on gas chromatography-mass spectrometry (GC-MS), liquid chromatography-mass spectrometry (LC-MS), or nuclear magnetic resonance (NMR) are commonly used^11, 12^. However, measurement of all these compounds is still an analytical challenge due to the complexity of the matrix samples^13^. In this regard, the use of hyphenated chromatographic techniques, such as comprehensive two-dimensional gas chromatography-mass spectrometry (GC × GC-MS), has emerged as a powerful separation technique to help solve this issue^14^.

The three main benefits of GC × GC compared to one-dimensional (1D) GC are: (i) increased chromatographic resolution; (ii) improved analyte detectability due to the cryofocusing in the thermal modulator; and (iii) chemical class ordering in the contour plots^15^. The coupling of two-dimensional (2D) GC × GC to a fast detector such as time-of-flight (TOF) working at acquisition rates up to 500 MHz and combined with a proper spectral deconvolution helps to fully resolve peak metabolites and enables the detection of up to several thousand peaks in a single GC × GC run^16, 17^. However, one problematic feature of metabolomic data is the complexity and large volume of data provided by GC × GC–TOFMS and the difficulty of analysing highly polar or thermal labile metabolites, which require complex derivatization reactions. Despite the technique’s increased resolution and the significant separation improvement over 1D GC – the 2D separation space provides large peak capacity – there is still some overlap^18–20^.

In the last decade, the generalised rank annihilation method (GRAM)^21^, parallel factor analysis (PARAFAC)^22^, PARAFAC2^23^ and the partial least squares (PLS)^24, 25^ multivariate resolution method have been proposed to overcome fundamental challenges occurring during GC × GC analyses. The most effective way to handle both elution time shifts and peak shape changes is to use methods that do not require the fulfilment of the trilinear model, such as the PARAFAC2 method^23, 26^ or, in more general cases, multivariate curve resolution-alternating least squares (MCR-ALS)^27–30^. As the MCR–ALS method is based only on fulfilment of the bilinear model’s assumption, three-way and four-way GC × GC–TOFMS data should be arranged in a column-wise super-augmented data matrix in which mass-to-charge ratios (m/z) are allocated in the columns and the elution times in the second and first chromatographic columns are listed in the rows^31, 32^. Since m/z values are common to all measured spectra in all second-column modulations, unavoidable chromatographic challenges, such as shifts in retention time within and between GC × GC–TOFMS chromatographic runs, are properly handled in the column-wise augmented mode. In addition, baseline/background contributions can be modelled by adding extra components to the MCR–ALS model. Another outstanding aspect of MCR–ALS analysis is its extreme flexibility to consider all samples (standard, unknown and replicates) in a single super-augmented data matrix, enabling joint analysis^30^, even in cases where the individual data matrices have different numbers of rows (retention times).

As mentioned above, the metabolic response of plants to various stresses of abiotic origin (e.g. metals, pesticides) is receiving increasing attention^33–35^. For instance, the occurrence of Cd and Pb have been shown to affect carbohydrate metabolism and glutathione metabolism, whilst crop exposure to herbicides has been shown to decrease antioxidant levels and disturb the tricarboxylic acid (TCA) cycle and other amino acid-related pathways^33, 34^. However, the effect of the presence of CECs in irrigation waters on plant metabolomics has not yet been considered. The aim of the present study was to conduct, for the first time, a non-targeted metabolomic analysis of lettuce (*Lactuca sativa* L) exposed to 11 CECs (benzophenone (BZP), bisphenol A (BPA), butylated hydroxytoluene (BHT), caffeine (CAF), carbamazepine (CBZ), methylparaben (MePB), 5-methyl-1H-benzotriazole (MeBT), 4-octylphenol (OPL), phenazone (PZE), triclosan (TCS) and tris(2-chloroethyl) phosphate (TCP)) supplied in irrigation water in order to determine the affected metabolic pathways. Extracted leaf samples were derivatised and analysed by GC × GC–TOFMS combined with MCR and PLS to identify the endpoints affected by the exposure to the contaminants.

## Results

### Occurrence and effect of CECs on plant morphology and physiology

**Figure 1:**
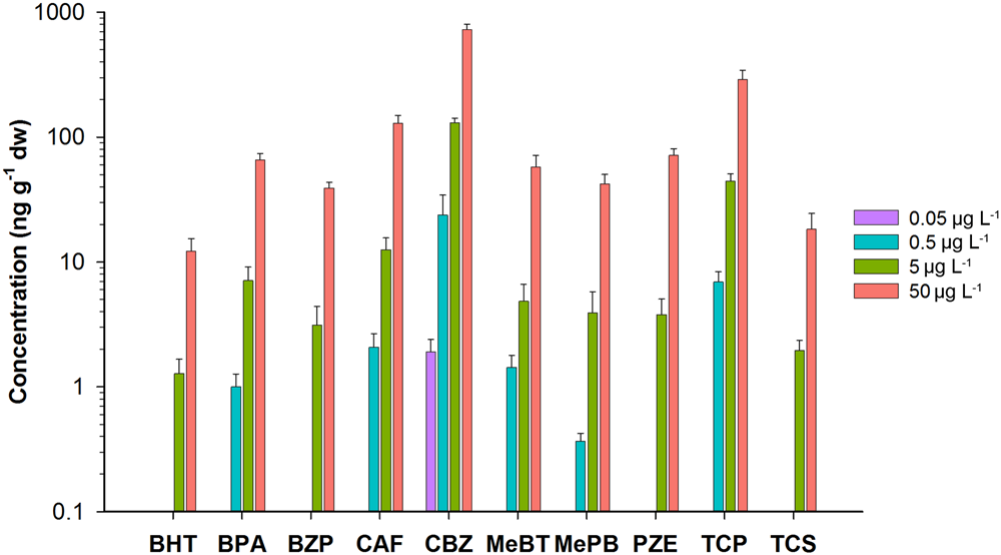
Mean concentration and standard deviation (N = 4) of the studied CECs in leaves (ng g^−1^ dw) for the four different irrigation concentrations (0.05, 0.5, 5 and 50 µg L^−1^) in log scale.

Figure 1 shows the concentration of the different CECs in the lettuce leaves of all the experimental units following an exposure period of 34 days. No CECs were detected in the non-exposed lettuce. At the lowest treatment concentration (i.e. 0.05 µg L^−1^), only CBZ was detected (2 ng g^−1^dw). At higher irrigation concentrations (i.e. 0.5 µg L^−1^), BPA, CAF, 5-MeBT, MePB and TCP were also detected (1–24 ng g^−1^dw). Finally, at the two highest treatment concentrations (i.e. 5 and 50 µg L^−1^), all the CECs except OP were detected (1–724 ng g^−1^dw). CEC concentrations increased over the course of the treatments in all cases. CBZ was the CEC to exhibit the highest accumulation in leaves, followed by TCP and CAF. BHT and TCS exhibited the lowest leaf accumulation. Linear correlation (Pearson correlation coefficient > 0.90; *p* < 0.05, N = 16) between the leaf and irrigation concentrations of CBZ was observed (Fig. S1).

**Figure 2:**
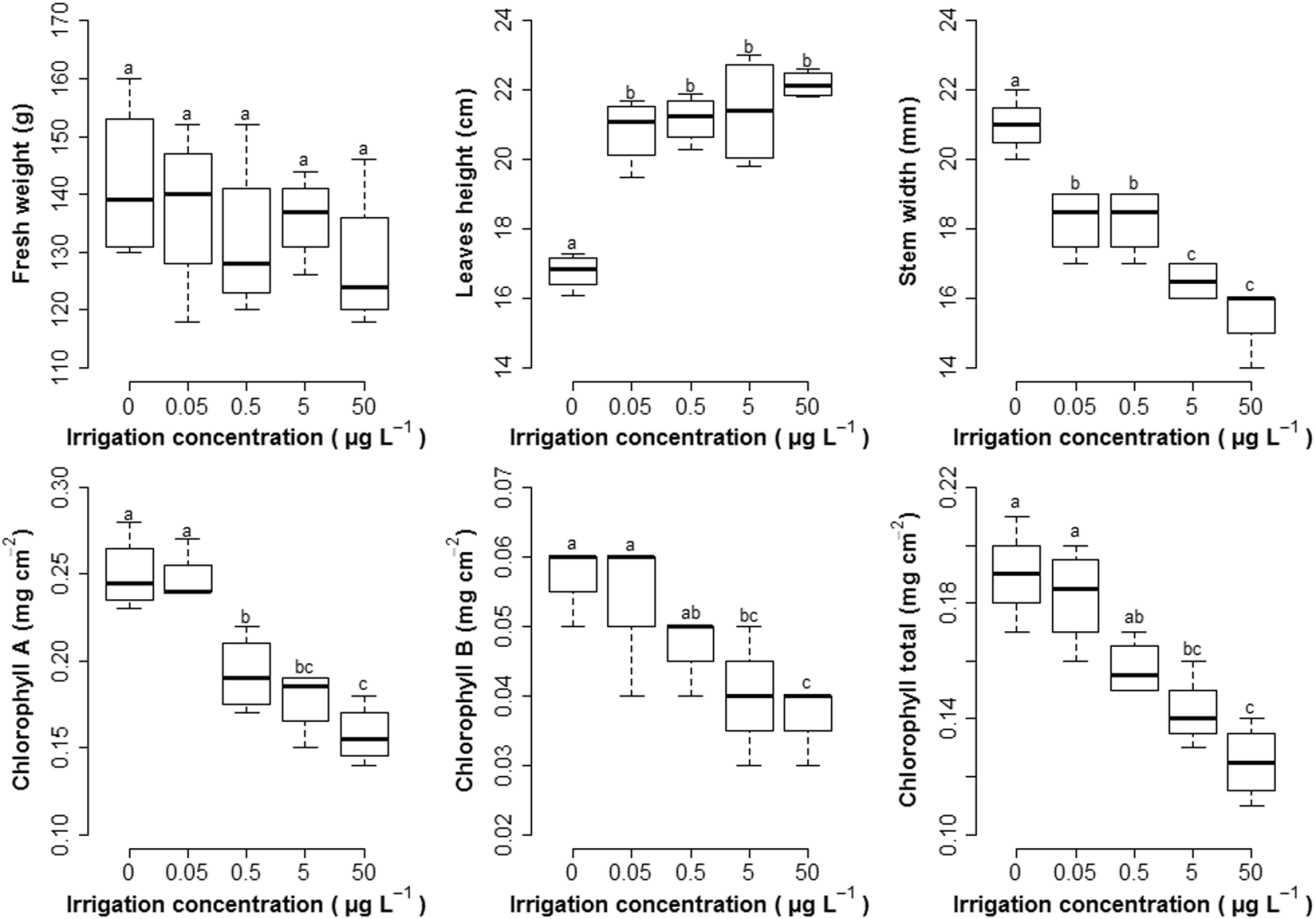
Boxplot of the studied agronomic parameters for the different irrigation concentrations (0, 0.05, 0.5, 5 and 50 µg L^−1^). Properties with the same letters are not significantly different (*p* < 0.05).

Figure 2 shows the effect of the presence of these CECs on plant morphology (fresh weight, leaf height and stem width) and physiology (chlorophylls A and B and total chlorophyll). Although there were visual differences in the lettuce exposed to different CEC concentrations, no significant differences (*p* > 0.05) were observed in the fresh weight. In contrast, differences in height were apparent between the exposed and non-exposed samples, although not between samples exposed to different concentrations. Differences in stem width were significant between exposed and non-exposed units and between different treatment levels. In addition, a significant reduction (between 15 and 37%) was observed in chlorophyll A and B content compared to non-exposed samples at different treatment levels (Fig. 2).

### GC × GC–TOFMS combined with MCR-ALS-PLS: a powerful tool for elucidating metabolic profiling

Data pre-processing with ChromaTOF software (LECO) revealed more than 1000 peaks (S/N ratio ≥ 100) in the lettuce extracts (Fig. S2). Several metabolites were identified, including sugars (e.g. glucose, galactose, ribose), alcohols (arabinitol and ribitol), and some organic acids (malic acid and gluconic acid). The highly overloaded peak at the 300–400 time point of the contour plot was assigned to fructose, the monosaccharide occurring at the highest concentration in lettuce (0.43% w/w)^36^.

Despite the unsurpassed GC × GC peak capacity, many plant components exhibited a strong coelution. Therefore, the application of chemometric tools was advisable to improve the resolution of the metabolite chromatographic profiles and their associated mass spectra for accurate metabolite identification. Raw GC × GC–TOFMS data from the 20 lettuce sample extracts were column-wise augmented and analysed by MCR-ALS applying the proper constraints (see the Supporting Materials (SM) section, Fig. S3 for further information)^27–32^. With 75 MCR-ALS components selected, R^2^ values (see Eq. S5) were higher than 99.8% and LOF values (see Eq. S6) were below 3.6%. Of these 75 MCR-ALS-resolved profiles, 50 were unambiguously assigned to characteristic lettuce metabolites by library search. These 50 MCR-ALS-resolved components were further considered for identification and linked to the lettuce metabolome.

Following the resolution and identification of the 50 lettuce metabolites, the peak areas of the same MCR-ALS component profiles in every sample and for all components and treatments were arranged in a data table. This data table with the 50 metabolite peak areas for the 20 lettuce samples (**X** data block) was then correlated to the 5 morphological and physiological plant parameter responses in the 20 lettuce samples (**Y** data block) using PLS2 (see Method section). The **X** and **Y** data blocks were auto-scaled before PLS2. Cross-validation (CV) was used to test the number of significant latent variables (LVs) needed in the PLSR correlation model in a practical and reliable way. Ultimately, 4 LVs were considered in the model. The cumulative captured variances were 85.5% for the X block and 79.8% for the **Y** block. Table S1 shows the explained variance by LV.

The variable importance in projection (VIP) scores represent the influence of each variable on the PLS model (Eq. S7). In other words, the VIP score for each variable was computed to quantify its importance using the PLS weights associated with each LV. The VIP scores were very useful in determining which of the metabolites detected in the lettuce extracts were most influential. Therefore, examination of the VIP scores is a reliable and simple technique for determining the effective metabolites in the best final PLS predictive model. Table 1 shows the VIP scores for the three morphological and physiological plant parameters of leaf height, stem width and total chlorophyll for 50 metabolites. In this study, the “greater than one rule” was generally used as a criterion for variable selection^37^. Therefore, **X**-variables with a VIP greater than one were important. With the aid of the VIP scores, it was possible to determine the most influential variables amongst a large number of variables in the **X** block. In this regard, 21 metabolites were found to significantly affect the four morphological and physiological parameters studied.

**Table 1:** VIP scores for the concentration of metabolites in lettuce crops in response to CEC stress exposure for the three morphological-physiological parameters measured: leaf height, stem width and total chlorophyll content.

Metabolite	Leaf height	Stem width	Total chlorophyll
**L-5-Oxoproline**	**2.42**	**3.39**	**3.20**
**L-Serine**	**1.40**	**1.84**	**1.75**
**3-Methyl-2-oxovaleric acid**	**1.35**	**1.29**	**1.31**
Adenosine	0.30	0.34	0.33
Phosphoric acid	0.51	0.54	0.53
γ-lactoneMannonic acid	0.20	0.03	0.05
Citric acid	0.92	0.39	0.49
Fumaric acid	0.64	0.12	0.24
**Galactonic acid**	**2.52**	**3.46**	**3.27**
**Gluconic acid**	**2.70**	**3.35**	**3.26**
Glyceric acid	0.85	0.90	0.90
**Malic acid**	**2.17**	**2.47**	**2.46**
Methylmalonic acid	0.32	0.04	0.09
Quinic acid	0.24	0.29	0.25
**Ribonic acid**	**2.03**	**2.94**	**2.76**
**Succinic acid**	**1.53**	**1.44**	**1.42**
Tartaric acid	0.64	0.13	0.24
**Threonic acid**	**0.97**	**1.00**	**0.97**
**2,3-Butanediol**	**1.89**	**1.65**	**1.75**
bis-1,2-acetin ether	0.68	0.09	0.21
**Allo-Inositol**	**1.77**	**1.83**	**1.74**
β-D-Galactopyranoside-(1,2)-glycerol	0.50	0.41	0.45
**Galactitol**	**1.99**	**2.26**	**2.19**
Glycerol	0.53	0.16	0.24
**Inositol isomer 1**	**2.23**	**2.74**	**2.59**
**Inositol isomer 2**	**2.24**	**3.15**	**2.97**
**Inositol isomer 3**	**1.96**	**1.82**	**1.76**
**meso-Erythritol**	**1.87**	**1.67**	**1.67**
Inositol isomer 4	0.58	0.50	0.46
Ribitol	0.43	0.12	0.19
(S,S,R,R,S)-methyl 6-deoxy-β-L-Galactopyranoside	0.91	1.34	1.25
2-O-Glycerol-α-d-galactopyranoside	0.87	1.04	0.99
**Allose**	**1.19**	**0.99**	**1.05**
Arabinose	0.81	0.43	0.55
Ethyl α-D-glucopyranoside	0.35	0.05	0.13
Galactose	0.46	0.60	0.57
**Glucopyranose**	**2.11**	**3.09**	**2.90**
**Glucose**	**1.85**	**1.60**	**1.67**
Lyxose	0.43	0.05	0.15
**Mannose**	**1.55**	**2.19**	**2.06**
Melibiose	0.31	0.05	0.13
Methyl-4-O-methyl-α-D-glucopyranoside	0.39	0.39	0.40
Ribofuranose	0.62	0.42	0.49
Ribose	0.42	0.33	0.36
**Sorbose**	**1.79**	**2.32**	**2.17**
Sucrose	0.46	0.09	0.15
Tagatofuranose	0.53	0.40	0.44
Tagatose	0.51	0.03	0.16
Trehalose	0.55	0.23	0.33
Xylose	0.61	0.75	0.68

Metabolites in bold indicate that VIP scores >1.

### Metabolic changes in response to the presence of CECs

Of the more than 1000 peaks detected by GC × GC–TOFMS (LECO’s ChromaTOF software) the mass of only 50 increased differently in lettuce leaves in response to the occurrence of CECs in irrigation waters (Fig. S2). The metabolites identified included amino acids, organic acids and sugars (Table S2).

**Figure 3:**
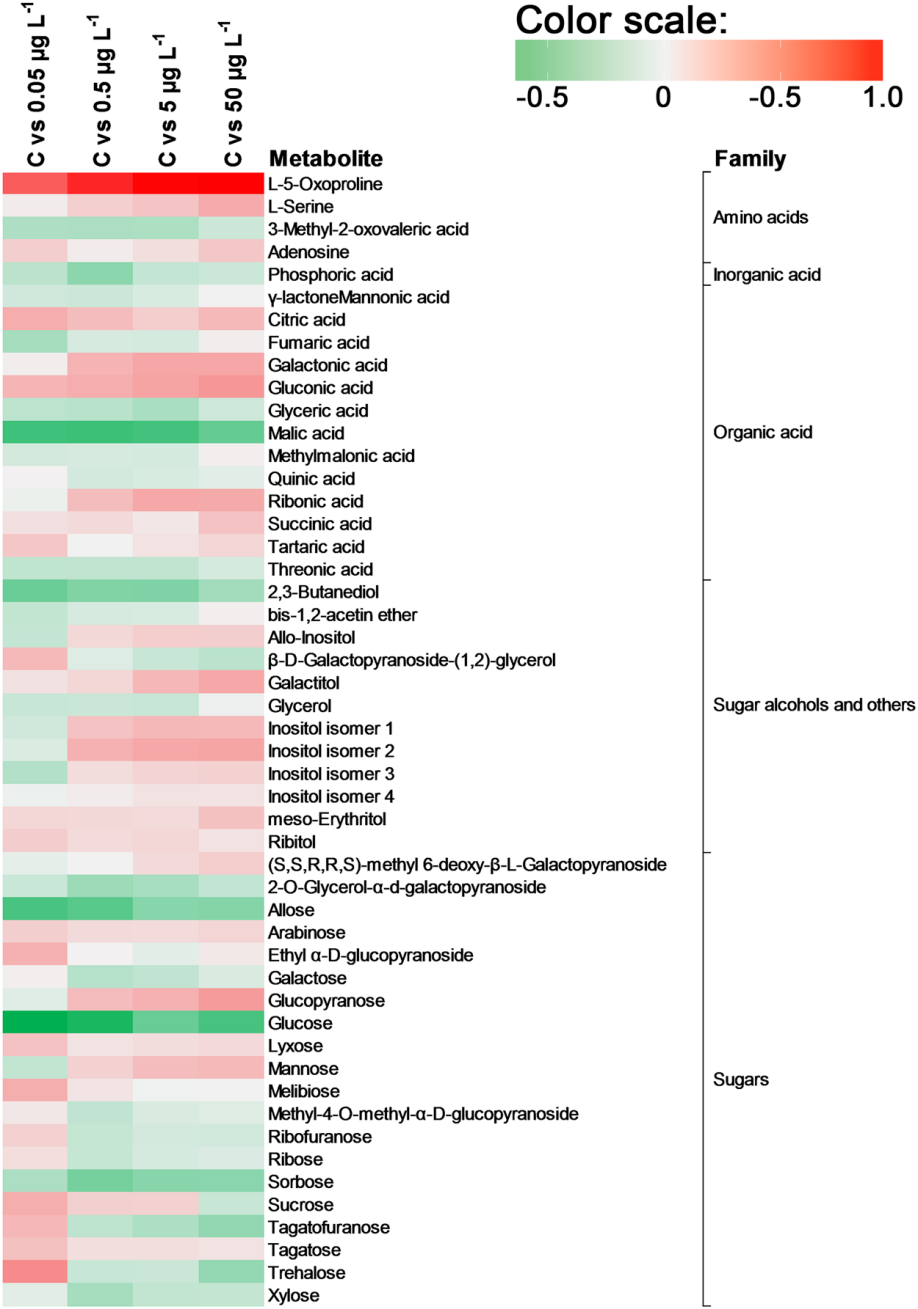
Heat map of changes in the levels of 50 metabolites in response to the CEC exposure concentration compared to unexposed lettuces (C). The log_10_ fold change in abundance ratios (see Eq. 1) are shown in Table S8. Red indicates an up-regulation effect, whilst green indicates a down-regulation effect.

The heat map showed up- and down-regulation of metabolites when exposed and non-exposed lettuces were compared (Fig. 3). One of the strongest effects was found for L-5-oxoproline, whose relative abundance increased in CEC-exposed lettuces in a dose-response manner (up-regulation). Differences in other amino acids, such as L-serine and adenosine, were observed between the control and exposed lettuces; however, the effects were lower than for L-5-oxoproline. More than 10 organic acids exhibited up- and down-regulation between treatments compared to the control samples. Malic acid was the organic acid to exhibit the greatest down-regulation when treated and control samples were compared. Threonic and glyceric acids were also found to be down-regulated when exposed and non-exposed samples were compared. In contrast, organic acids such as gluconic acid or galactonic acid, were up-regulated (log ratio > 0.2). Many carbohydrates (e.g. sorbose or allose) exhibited down-regulation. Nevertheless, some sugars were up-regulated by the presence of CECs, such as mannose (up to 0.23) or glucopyranose (up to 35). In fact, there is evidence that plants respond to environmental factors through sugar-sensing mechanisms^38^. Finally, many alcohols and other compounds, such as inositol isomers and 2,3-butanediol, also exhibited significant up or down-regulation respectively.

### Morphological changes and metabolic pathways impaired by the presence of CECs in irrigation waters

Interestingly, even exposure to the lowest CEC concentration (0.05 µg L^−1^) affected the lettuces’ metabolite profiles. The amino acid 5-oxoproline, organic acids (i.e. malic acid, glucuronic acid, ribonic acid and galactonic acid), sugars (i.e. glucopyranose) and sugar alcohols (i.e. inositol isomers, galactitol) showed the highest correlation with plant morphological and physiological responses.

**Figure 4:**
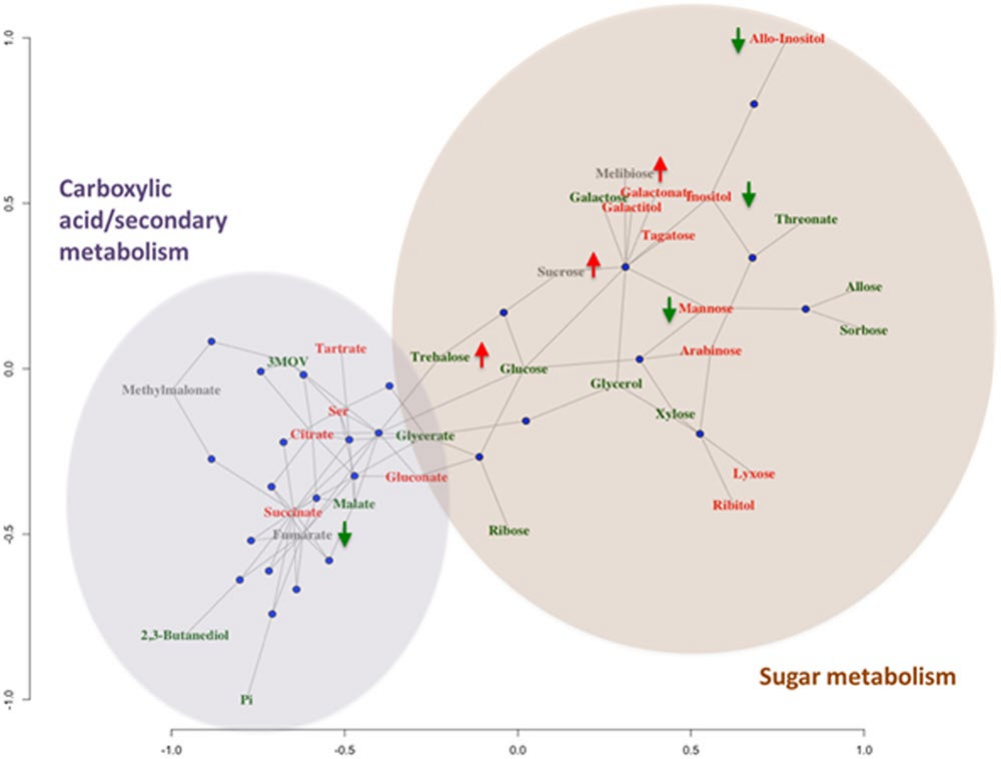
KEGG pathway analysis of the analyzed metabolites, using *A. thaliana* pathway dataset (Table S3). Two functional groups can be observed at the bipartite plot (carboxyclic acid/secondary metabolism and sugar metabolism).

KEGG pathway analysis of the analysed metabolites using the *A. thaliana* pathway data set identified 29 functional modules that included at least two metabolites (Table S1). Pathways related to secondary metabolism (ath01110, ath01200, ath00630, ath00020) and sugar metabolism (ath0052, ath00040, ath00030, ath00520) were especially well-represented, probably indicating an effect of the treatment on these two functions (Table S3). These two functional groups can be observed in the bipartite plot shown in Fig. 4. The graph also shows the coordinate reduction of several saccharides (glucose, galactose, ribose, trehalose, allose, sorbose, and xylose, labelled in green in Fig. 4) and the increase in some of their metabolites (gluconate, ribitol, galactitol, and galactonate, labelled in red in Fig. 4), which may indicate an increase in the catabolism of non-structural carbohydrates. The graph likewise shows an increase in the inositol pathway. Arrows in the plot indicate metabolites that show a differential response at the lowest treatment concentration (see also the heat map in Fig. 3); it is revealing that strong and mild treatments had virtually opposite effects on several sugars and on the inositol pathway.

The secondary metabolism cluster is centred on four TCA cycle metabolites (citrate, succinate, malate and fumarate), although it is unclear whether or not these changes can affect the plant’s energy metabolism capacity, as none of the other related functional modules appeared to be affected. The connection of these metabolites with other pathways related to secondary metabolites (2,3-butanediol, tartrate, glycerate) may suggest an effect on the plants’ secondary metabolism, possibly related to their defence mechanisms, as occurs with embryos exposed to BPA^39–43^.

**Figure 5:**
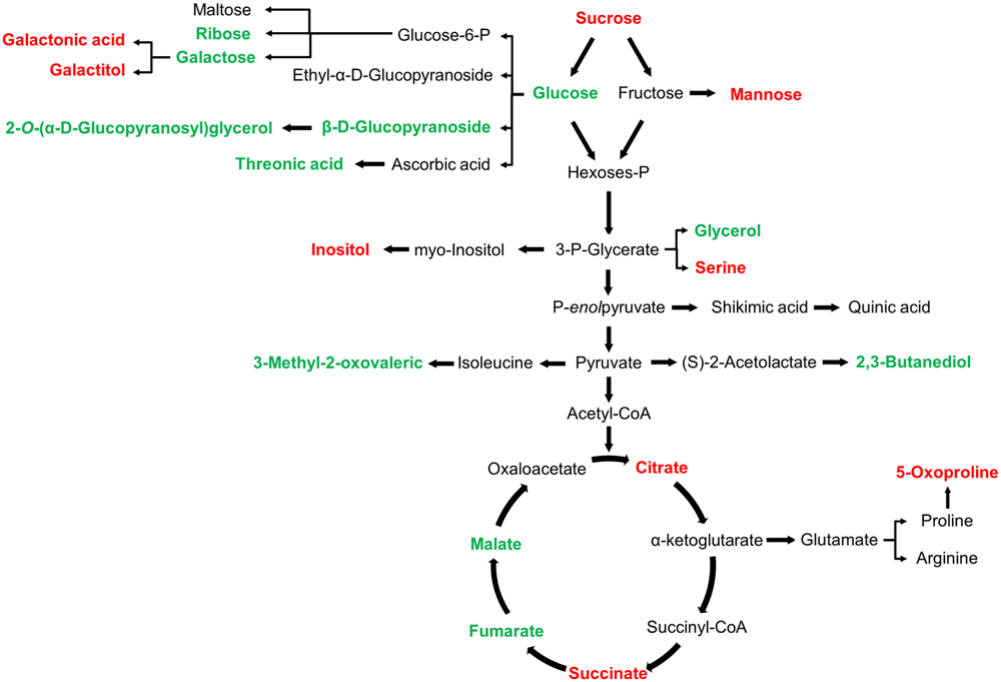
Metabolic changes involved in the primary pathways of lettuce exposed to CECs. The significant up and down regulated metabolites are indicated in red and green respectively.

A comparison of the metabolite clusters shown in Fig. 4 and the calculated PLSR model and VIP scores revealed that 21 metabolites significantly impaired the four plant morphological and physiological parameters studied. These metabolites included those involved in galactose metabolism (e.g. mannose, galactitol and inositol), three of the metabolites involved in the TCA cycle (succinate, malate and fumarate), and other components of sucrose metabolism, glycerolipid metabolism, the pentose phosphate pathway and the amino acid pathways (Fig. 5).

## Discussion

Plant uptake of CECs by the lettuce crops was consistent with previous findings in which neutral compounds, such as carbamazepine, have been shown to be prone to uptake in crops^44–46^. The relationship between the CEC concentrations in leaves and irrigation water was only fairly linear for CBZ, which could be detected at the four assessed concentrations. Therefore, bioaccumulation factors (concentration ratio in leaves in dry weight vs irrigation concentration) were CEC dependent. CBZ and TCP exhibited the highest values (14 and 5.7 L kg^−1^ dw respectively), whilst BHT and TCS exhibited the lowest values (0.24 and 0.36 L kg^−1^ dw respectively). These values were similar to the values reported in the literature for different experimental setups^45–49^. Ionic and hydrophobic (BPA, TCS) CECs exhibited lower translocation than non-ionic and polar compounds (CBZ and TCP), in keeping with previously reported findings^50, 51^.

Metabolomic studies require powerful analytical tools. In the present study, the use of GC × GC–TOFMS proved to have a high separation capacity for complex samples, resulting in the detection of over 1,000 peaks with a S/N ration > 100. The main limitations are the thermal stability of analytes during GC analysis and the large size of the database when TOFMS raw data are processed. The present study demonstrated that chemometric tools such as MCR-ALS followed by PLS could be used to compare the metabolic profiles of different samples.

With regard to the effect of CECs on plant physiology and morphology, recent findings have proved that the presence of CECs produces changes in plant hormone concentrations (e.g. auxins, cytokinins, abscisic acid and jasmonates), the nutrient composition of crop leaves^52^, biomass production, chlorophyll content and plants’ shoot growth^53–56^. However, these studies were carried out exposing plants at several orders of magnitude (i.e. mg L^−1^ levels) higher than the relevant environmental concentrations. The results of the present study expand on those findings, confirming that crop exposure to CECs affected morphological and physiological parameters, such as the leaf height, stem width and chlorophyll A and B content of lettuce, at the relatively low concentration of 0.05 µg L^−1^. This is the first time that plant exposure to relevant environmental concentrations of CECs has been shown to affect plant morphology and physiology.

Primary metabolism plays an essential role in plant metabolism and is vital for plants’ survival and development^57^. The present study explored the metabolic response of lettuce crops exposed to different CEC concentrations (Fig. 3). Carbon (C) and nitrogen (N) metabolism are closely coordinated in the fundamental processes permitting plant growth, e.g. photosynthesis and N uptake^58^. Figure 3 shows that CEC concentrations play an important role in plants’ metabolic responses. The response is therefore normally greater (either up- or down-regulated) at higher concentrations (e.g. 5-oxoproline and gluconic acid). However, for most of the metabolites (e.g. mannose, glucose, malic acid and allose) the dose-response relationship was non-linear (Fig. 3). This is in keeping with the fact that at low doses organic pollutants such as herbicides (e.g. glyphosate and simazine) stimulate plant growth or protein content, whereas at higher doses they cause phytotoxicity; indeed, such hormetic responses are not unusual in (eco)toxicology^59^.

The metabolites can be classified as up-regulated (5-oxoproline, serine, adenosine, citric acid, galactonic acid, gluconic acid, ribonic acid, succinic acid, glucopyranose, mannose, inositols, galactitol) and down-regulated (allose, glucose, ribose, tagatofuranose, trehalose, xylose, malic acid, butanediol). The amino acid 5-oxoproline showed the highest up-regulation response to CEC exposure (Fig. 3). This is consistent with the fact that this metabolite is an intermediate of the metabolism of glutathione, one of the main plant defence substances^60^. It is involved in many detoxifying mechanisms, such as the reduction of active oxygen species, and also regulates cell defence systems, including the detoxification of toxic xenobiotics, such as herbicides^61^. Other metabolites up-regulated by the presence of CECs include amino acids (serine and adenosine), organic acids (citric acid, galactonic acid, gluconic acid, ribonic acid and succinic acid), sugars (glucopyranose and mannose), sugar alcohols and others (inositols and galactitol). The amino acid serine is also involved in the glutathione response through the stabilisation of the glutathione thiolate anion^62^. Citric acid is associated with oxidative stress tolerance to heavy metals^63^. Similarly, a concentration increase in gluconic acid has been reported in plants exposed to different metal elements (i.e. Cd and Pb)^33, 64^. Wang *et al*.^33^ reported that under the stimulus of Cd stress, carbon flow in radish roots mainly concentrated in gluconic acid, suggesting that it was being used to alleviate the Cd toxicity. Moreover, myo-inositol has been observed to be very strongly stimulated by glyphosate at doses above 10 or 40 μM, depending on the plant^65^. Recent studies in *Arabidopsis* suggest that nuclear pools of myo-inositol may play a specific role in programmed cell death. In fact, the regulation of myo-inositol levels is critical to maintain levels of ascorbic acid^66^, phosphatidylinositol, and ceramides, which regulate growth, development and cell death^67^. In contrast, sugars (e.g. allose, glucose, ribose, tagatofuranose, trehalose and xylose), malic acid and butanediol were the metabolites to show the highest down-regulation response to CEC exposure. The sharp decline in glucose after plant exposure to CECs indicates a shift from C accumulation to C assimilation^38^. Non-structural sugars play many roles in plant physiology, not only as energy reserves, but also as protection against osmotic pressure and other forms of stress^68^. Glucose concentration has been observed to decline in plants exposed to several pesticides, including glufosinate, sulcotrione, AE 944 [N2-(1-ethyl-3-phenylpropyl)-6-(1-fluoro-1-methylethyl)-1,3,5-triazine-2,4-diamine], foramsulfuron, benfuresate and glyphosate^69^. Additionally, the lower amounts of central C metabolism intermediates (glycolysis and TCA cycle) observed in lettuce crops exposed to CECs is likely associated with increases in energy consumption^70, 71^ (Fig. 5). These results are also consistent with a previous study showing down-regulation of malic acid during Pb stress in radish^33^. Finally, butanediol has been described as a signalling molecule involved in plant/bacterium interactions and, notably, able to induce plant systemic resistance^72^. The decrease in this metabolite under the occurrence of CECs may suggest a reduction of plant resistance to bacterial infection.

Changes in the metabolic profile could be linked to morphological parameters, such as plant elongation or chlorophyll content. In this study, the increase in the levels of 5-oxoproline and sugar metabolites (i.e. gluconic acid, glucopyranose, inositol isoforms, ribonic acid, galactitol and galactonic acid) was observed to be associated with an increase in leaf height and a decrease in stem width and total chlorophyll content (Table 1, VIP scores >2). Therefore, the occurrence of CECs may be associated with an increase in 5-oxoproline from the TCA cycle and sugar metabolism, which is related with plant cell elongation. For example, Zhao *et al*.^70^ observed that an increase in 5-oxoproline, amongst other metabolites, was associated with cell elongation. Proline biosynthesis deficiency leads to abnormal plants and cell wall defects, suggesting that it plays a role in structural proteins, some of which modulate stem elongation and shoot growth^73^. In contrast, a decrease in the level of malic acid was associated with increased leaf height, but decreased stem width and chlorophyll content. This is in keeping with the fact that foliar application of malic acid significantly increases chlorophyll content in *Lilium* sp. plants^74^ and that the malic acid concentration has been observed to increase during rapid cell elongation in cotton plants^75^. The malate valve uses malic acid to transport nicotinamide adenine dinucleotide phosphate (NADPH) generated by photosynthesis between cell compartments (chloroplast, cytosol and mitochondria)^76, 77^. Therefore, the decrease in chlorophyll content observed in the present study suggests an association with a reduction in photosynthesis and, consequently, a reduction in the malic acid needed to transport NADPH. Whilst malic acid could not be utilised, the oxidative pentose phosphate pathway (PPP) may serve as a major non-photosynthetic source of NADPH production, which is needed for the reduction of oxidised glutathione^78, 79^ due to CEC exposure. Accordingly, the observed increase in gluconic acid (Fig. 3), an intermediate metabolite of PPP, could be the response to CEC exposure.

In summary, morphological and metabolic analysis of lettuce crops irrigated with CECs demonstrated that the presence of these compounds in irrigation waters disrupted carbohydrate metabolism, including of glucose, inositol, sorbose, mannose and allose, the TCA cycle (e.g. malic acid, fumarate, and citric acid), the pentose phosphate pathway (gluconic acid), and the glutathione pathway (5-oxoproline and serine) at all levels (Figs 4 and 5). Proline metabolism is apparently the most disrupted pathway, which may be explained by an enhancement of the glutathione detoxification pathway. These metabolic changes were associated with morphological (leaf height, stem width) and physiological (chlorophyll content) changes. According to the therapeutic doses, the concentration at which CECs have been found in lettuce crops do not pose a human health risk^79^. Nevertheless, the present results show that vegetable quality is affected by, amongst other things: metabolites (sugars, amino acids and carboxylic acids) and morphological-physiological parameters (leaf height, stem width and total chlorophyll content). The profile alteration of these metabolites may result in CEC-exposure-induced changes in flavour and nutritional value. Further studies at the genomic, transcriptomic and proteomic levels need to be performed to understand the mechanisms involved in the plant response to CECs.

## Methods

### Selection of crops and CECs

Lettuce (*Lactuca sativa* L) is an economically important vegetable crop that alone accounts for 14% of all leafy vegetable sales in the US^80^. The CECs were selected on the basis of their physicochemical properties spanning 4 orders of magnitude of lipophilicity, high recalcitrance (carbamazepine), and moderate acidity-basicity so that the neutral form, which is the most prone to uptake by passive membrane diffusion processes, would predominate at the soil pH. The tested concentration was in the range of the concentrations found for these compounds in surface water bodies and reclaimed water^81^. The CECs measured in the experimental study are listed in Table S4 in the Supporting Materials (SM) section.

### Experimental setup

The experiment was conducted in an agricultural experimental station (Agròpolis) belonging to the Polytechnic University of Catalonia (UPC, Viladecans, Spain) in winter (29 January to 21 March 2016). The average temperature inside the greenhouse was 21 °C (8 °C min and 29 °C max) and the relative humidity was 56% (32% min and 78% max). Experimental units consisted of 2.5 L cylindrical amber glass pots (Ø = 15 cm and 20 cm high) fitted with a bottom outlet connected to drainage tubing (Ø = 3 cm) and filled with 2.3 kg of air-dried soil sieved to 2 mm. The soil used was collected from the surface horizon of a Typic Xerorthents soil from an agricultural area located in the Llobregat River Delta (longitude 2°03′E, latitude 41°17′N). The soil had a sandy texture (90% sand, 8% silt, 2% clay), a pH of 7.4 (soil-to-water ratio 1:5) and an electrical conductivity of 3.8 dS m^−1^ (soil-to-water ratio 1:5). The total organic carbon content was 5 g kg^−1^ and the total organic nitrogen content was 0.7 g kg^−1^. The cation exchange capacity (CEC) was 3.8 meq. 100 g^−1^ and exchangeable Ca^2+^, Mg^2+^, Na^+^ and K^+^ were 2.82, 0.64, 0.25 and 0.15 meq. 100 g^−1^ soil, respectively.

Lettuce (*Lactuca sativa* L, cv. Arena) was planted in pots and watered with Hoagland nutrient solution^82^ diluted 1:1 with rainwater. About 100 mL of irrigation water was applied to each experimental unit per day. The number of daily irrigations was regulated to keep water in the soil below field capacity, thereby preventing leachate production. The CECs were added to the soil 18 days after the seedling implant. Five treatments were used, consisting of the direct application of 0, 0.05, 0.5, 5 and 50 μg L^−1^ to the irrigation water in four replicates per treatment (20 experimental units in total). After 34 days, the lettuce was harvested and the leaves were comminuted separately with liquid nitrogen and stored at −80 °C until analysis.

### Sample preparation

#### CEC extraction from the lettuce leaves

The extraction procedure for the determination of the CECs in the leaves is described elsewhere^83^. For each experimental unit, a 0.5 g portion of the comminuted leaves was transferred to a porcelain mortar and spiked with a mixture of surrogates (see SM section). Briefly, a matrix solid-phase dispersion (MSPD) method was performed, followed by a pressurised solvent extraction using a mixture of acetone:hexane (1:1, v/v). The final extracts were analysed by GC coupled to electron impact tandem mass spectrometry (EI-MS/MS) in a Bruker 450-GC gas chromatograph coupled to a Bruker 320-MS triple-stage quadrupole mass spectrometer (Bruker Daltonics Inc., Billerica, MA, USA). Qualitative and quantitative analyses were performed based on retention time and the selected reaction monitoring (SRM) mode of two product ions. The ratio between the product ions was used for confirmation. The monitoring ions, limits of detection (LODs), limits of quantification (LOQs) and recoveries can be found in the SM (Tables S5–S7). The mean concentration and standard deviation of each CEC in the different treatments were then calculated with the four replicates of each treatment.

#### Metabolite extraction from the lettuce leaves

The extraction procedure was adapted from a procedure described elsewhere^84^. Briefly, for the extraction of polar plant compounds, 60 mg of plant material was transferred into an Eppendorf tube. Then, 400 µL methanol, 30 µL of a 50 µg mL^−1^ D-glucose (U-^13^C_6_, 99%) solution and 30 µL of a 50 µg mL^−1^ salicylic acid-d_6_ solution were added to the tube. The samples were vortexed and sonicated in an ultrasonic bath for 15 min. 200 µL of chloroform was then added and the samples were vortexed for 1 min and sonicated for 15 min. Next, 400 µL of water was then added and the samples were again vortexed for 1 min and sonicated for 15 min. The tubes were then centrifuged at 10,000 × *g* for 15 min in order to separate the aqueous and lipid phases. Finally, 700 µL of the aqueous phase was transferred to a 4 mL glass vial. The extracts were vacuum-dried with a Speedvac (Thermo Scientific) at 40 °C for 4 h. The samples were stored at −80 °C until analysis. 200 µL of 20 mg mL^−1^ methoxyamine (MeOX) in pyridine was added to the dry residue. The mixture was vortexed for 1 min and then incubated at 40 °C for 90 min. Thereafter, 30 µL of *N*-methyl-*N*-(trimethylsilyl) trifluoroacetamide (MSTFA) with 1% trimethylchlorosilane (TMCS) was added and the mixture was vortexed for 1 min and incubated at 40 °C for 45 min. Finally, the extracts were filtered through a 0.22 µm filter (Ultrafree®-MC, Millipore) and then transferred to a chromatographic vial. Triphenylamine (TPhA) was added as an instrumental standard (25 µL) and samples were injected into the GC × GC-TOFMS.

#### Instrumental analysis

The GC × GC-TOFMS system consisted of an HP 6890 N (Agilent Technologies, Palo Alto, CA) gas chromatograph equipped with a split/splitless injector, a secondary oven to fit the secondary column, and a ZX1 (Zoex, Houston, TX) two-stage thermal modulator operating at 4 s per modulation with 0.5 s hot pulse duration and a 30 °C modulator temperature offset. Liquid nitrogen was used to cool down the nitrogen gas for cold pulses and was automatically filled into a Dewar using a liquid leveller, which accessed a 60 L liquid nitrogen storage tank. For the first dimension, a 20 m × 0.18 mm I.D., 0.36 µm film thickness Sapiens-X5.MS coated with 5% diphenyl 95% dimethyl polysiloxane from Teknokroma (Sant Cugat del Vallès, Spain) was used. For the second dimension, a 2 m × 0.10 mm I.D., 0.10 µm film thickness TRB-50HT from Teknokroma was used. The oven temperature was held at 75 °C for 2 min and then programmed to rise at 7 °C min^−1^ to 100 °C, then at 5 °C min^−1^ to 260 °C, and finally at 10 °C min^−1^ to 310 °C, with the final temperature being held for 5 minutes. The secondary oven was kept 5 °C above the first-dimension temperature throughout the chromatographic run. Helium was used as the carrier gas at a constant flow of 0.6 mL min^−1^. In addition, 1 µL of each sample was injected via the autosampler in splitless injection mode. The MS system was a low resolution Pegasus 4D TOF system (LECO, St. Joseph, MI) operating in the electron impact ionisation mode. The applied electron energy was 70 eV, and the transfer line and ion source were set at 250 °C and 200 °C, respectively. Scanning was performed from 60 to 700 m/z at 100 spectra s^−1^ with unit mass resolution and a detector voltage of 1800 V. The data was pre-processed in Chroma-TOF 3.32 software using the peak find and peak and spectra deconvolution software routines for the identification and annotation of detected peaks. A signal-to-noise ratio of 100 was used for this study.

### Data processing and statistical analysis

#### Agronomic parameters

Morphological (fresh weight, leaf height and stem width) and physiological (chlorophyll A and B) parameters were measured after 34 days. For the determination of chlorophyll A and B, three circles of inner, middle and outer leaf were cut using a circular cutting press (4 cm diameter per circle, 12.6 cm^2^ foliar area) and individually extracted with 5 mL N,N-dimethylformamide (DMF)^85^. Tubes were kept from the light and stored at 4 °C for 48 h. UV absorbance at 647 and 664.5 nm was then measured using a Varian Cary 400 spectrophotometer (Agilent Technologies, CA, USA). Chlorophyll concentration was calculated based on the absorbance values and the foliar area as described in the SM section (Eqs S1–S3). Mean values for the three circles of each experimental unit were used to calculate the average chlorophyll content of each treatment.

#### Data arrangement, compression and MCR-ALS analysis

Data sets were in.CDF format and were imported into MATLAB using MATLAB’s Bioinformatics Toolbox. Data from GC × GC-TOFMS analyses can be arranged in a three-way data cube or array with two retention time axes and one m/z values axis. If more than one sample is analysed, the data will be a four-way data array with two retention time axes, one m/z values axis and one sample axis.

Due to the huge amount of data collected in the GC × GC-TOFMS data sets for the 20 lettuce samples, a data segmentation and compression strategy based on the use of wavelets^86, 87^, especially in the time direction, was proposed to make their chemometric analysis more feasible and reduce computer storage requirements. To this end, GC × GC-TOFMS data for the 20 samples were segmented into four parts (A–D) by visual inspection of the chromatograms (Fig. S4). The bleeding part of the chromatogram was excluded from the data.

Since the same m/z range (i.e. 60–700 amu with 640 m/z points) was selected for all the chromatographic runs, GC × GC-TOFMS data for segments A–D for the 20 lettuce samples were arranged in a column-wise super-augmented matrix with their m/z values in the common column mode. In this column-wise augmented matrix arrangement, the same number of m/z values was observed for all modulations, whereas the number of elution times considered in each data modulation could be different. Thus, components common to different modulations can be described by elution profiles (peaks) with different shapes and retention times, even if they belong to the same compound in different modulations. This cannot generally be done with methods such as PARAFAC or PARAFAC2^27, 30, 88, 89^.

Since GC × GC-TOFMS produces a series of modulated peaks for each component, summation of the modulated peaks for each component can be used for quantitative analysis. MCR-ALS can resolve pure modulated peaks (second-dimension elution profiles) in the presence of baseline/background contribution and other chemical constituents. Relative quantitative information for one target compound can then be directly derived from the comparison of MCR-ALS-resolved second-dimension elution profiles for different samples. After resolving the GC × GC-TOFMS data for the 20 samples and obtaining the resolved elution profiles in two chromatographic columns along with their mass spectra, the lettuce metabolites were quantified and identified. These pure spectra can be used to identify the resolved components by comparing them with those of standard compounds in the National Institute of Standards and Technology (NIST) MS and Golm Metabolome databases. A reverse match factor (RMF) based on the correlation coefficient between the MCR-ALS-resolved and experimental mass spectra reported by the NIST software was used to select the best identified compound for the MCR-ALS-resolved mass spectra. This match factor is reported between 0 (no match) and 1000 (perfect match). As a general guide, a value of 900 or greater was considered to be a very good match; between 800 and 900, a good match; between 700 and 800, a fair match; and less than 600 a poor or very poor match.

#### PLS modeling

The relative concentrations of the identified metabolites were obtained using simple peak integration of resolved elution profiles in the second chromatographic dimension for each component. These concentrations were used to build a new matrix containing relative concentrations of all the resolved metabolites for the 20 lettuce samples (control and exposed). This new data matrix was then correlated to agronomic parameters obtained for the 20 samples including leaf height, stem width, chlorophyll A concentration, total chlorophyll concentration and soil pH by the PLS2 multivariate regression method to find the most discriminant metabolites for each agronomic parameter. For a straightforward interpretation of the PLS2 model, VIP^37^ scores were used instead of the commonly used regression coefficient vectors. VIP scores give information about how the variables combine to form the quantitative relation between **X** and **Y**, thereby providing a better assessment of their relative importance in the model. Hence, these VIP scores are useful for understanding which **X**-variables are important (numerically large VIP-score values) and which **X**-variables provide the same information (similar profiles of VIP-score values). A large VIP-score value in a chromatographic region indicates that the compounds eluted in that retention-time region will have a large impact on the prediction model, whilst a low value indicates that the components are less influential.

#### Heat map

To study the change in metabolites between the non-exposed lettuces (control samples) and the lettuces exposed to the four different concentrations, the ratio of the areas between the exposed and non-exposed lettuces was calculated. The logarithms of the means for each treatment were then used to plot a heat map (Table S8) for the different metabolites. Log ratio values above 0 indicate an up-regulation of metabolites in the exposed samples compared to the control (non-exposed) samples. Conversely, log_10_ ratio values below 0 indicate a down-regulation of metabolites in exposed units compared to the control ones.

1 $$\mathrm{log}\,ratio=\,\mathrm{log}(\frac{{\bar{A}}_{i}}{{\bar{A}}_{c}})$$

where ${\bar{A}}_{i}$ is the average abundance of a selected metabolite in lettuce samples exposed to different CECs and ${\bar{A}}_{c}$ is the average abundance of a selected metabolite in the control samples.

#### Metabolite and pathway identification

After the metabolites were identified using the NIST database, they were characterised with the Kyoto Encyclopedia of Genes and Genomes (KEGG) database. The same database was used to investigate the possible metabolic pathways involved^90, 91^.

The KEGG pathway analysis tool was used with the *Arabidopsis thaliana* database was used to construct an incidence table in which any given pair of metabolites was considered related if they appeared in at least one common pathway (Table S3). Identified pathways with at least two hits were included in a network analysis, using the reshape2 and igraph packages in R^92^. Only pathways with at least two identified metabolites were included in the analysis. The general pathways ath01100 (Metabolic pathways) and ath02010 (ABC transporters) were excluded from the analysis.

#### Software

GC × GC-MS data were acquired using ChromaTOF version 3.3.2 (LECO, St. Joseph, MI, USA), converted to CSV format in the 60–700 m/z range and imported into MATLAB using MATLAB’s Bioinformatics Toolbox (The Mathworks, Inc., Natick, MA, USA). NIST MS Search version 2.2 (National Institute of Standards and Technology, USA) and the GOLM metabolome database (GMD) of derivatised compounds were used for metabolite identifications. MATLAB’s wavelet toolbox was used for data compression. The PLS toolbox (http://www.eigenvector.com/) and MCR-ALS toolbox were used for chemometric analysis^93^.

## Electronic supplementary material

Supplementary Materials
