# Supplementary material for: Linking the morphological and metabolomic response of Lactuca sativa L exposed to emerging contaminants using GC × GC-MS and chemometric tools
Source: Sci Rep. 2017 Jul 26;7:6546. doi: 10.1038/s41598-017-06773-0 (PMC5529569; doi:10.1038/s41598-017-06773-0)
Supplement: Supplementary file 1 — Supplementary Materials [file 41598_2017_6773_MOESM1_ESM.pdf]

Supporting Materials for

Linking the morphological and metabolomic response of *Lactuca sativa* L exposed to emerging contaminants using GC×GC-MS and chemometric tools

Carlos Hurtado[1], Hadi Parastar[2], Víctor Matamoros[1], Benjamín Piña[1], Romà Tauler[1] & Josep M Bayona[1*]

[1]*Department of Environmental Chemistry, IDAEA-CSIC, c/Jordi Girona, 18-26, E-08034, Barcelona, Spain*

[2]*Department of Chemistry, Sharif University of Technology, Tehran, Iran*

*Correspondence to josep.bayona@idaea.csic.es

Method section

CECs and reagents

Benzophenone (BZP, 99%), bisphenol A (BPA, 99%), butylated hydroxytoluene (BHT, 98%) caffeine (CAF, 99%), carbamazepine (CBZ, 99%), methyl paraben (MePB, 99%), 5-methyl-1*H*-benzotriazole (MeBT, 98%), 4-octylphenol (OPL, 99%), phenazone (PZE, 98%), triclosan (TCS, 97%) and tris(2-chloroethyl) phosphate (TCP, 97 %) and all were purchased from Sigma-Aldrich (St. Louis, MO, USA).

Florisil was purchased from Merck (Darmstadt, Germany). Anhydrous sodium sulfate (Na_2SO_4) and sodium chloride (NaCl) were purchased from Fluka (Buchs, Switzerland). Disodium hydrogen citrate sesquihydrate and trisodium citrate dihydrate were obtained from Sigma-Aldrich. Suprasolv® grade acetone, methanol, hexane, ethyl acetate and LiChrosolv® grade acetonitrile were purchased from Merck. Hydrochloric acid (37% v/v) and potassium carbonate (98 %) were purchased from Panreac (Barcelona, Spain). The Na_2SO_4 was baked for 5 hours at 450 ºC in a muffle furnace before using. Reagent water was deionized in the laboratory using the ultrapure water system Arium 611 from Sartorius (Aubagne, France).

D-glucose (U-13C6, 99 %) was supplied by Cambridge Isotope Laboratories, Inc. (Andover, MA, USA), salicylic acid-d6 (98% D), triphenylamine (TPhA) and trimethylsulfonium hydroxide (TMSH) were purchased from Sigma Aldrich. Pyridine (anhydrous, 99.8 %), chlorotrimethylsilane (TMCS), methoxyamine hydrochloride (98 %) (MeOX) and N-methyl-N-trimethylsilyl trifluoroacetamide (>98.5 %) (MSTFA), used as derivatizating agents, were also obtained from Sigma-Aldrich. Hexane, methanol and chloroform were analytical reagent grade, and sodium chloride (NaCl) salt was supplied by Merck (Darmstadt, Germany). N,N-dimehtylformamide (DMF) was purchased to Sigma-Aldrich.

CEC extraction from the lettuce leaves

Leaf tissue spiked with a mixture of surrogates was extracted with a mixture of acetone: hexane (1:1, v/v) using a pressurized solvent extraction (PSE) system (Applied Separations, PA, USA). Neutral-basic and acid fractions were obtained by solvent partitioning at neutral and acid pH, respectively. After a cleanup with Florisil and sodium sulfate, TPhA was added as internal standard and TMSH was added as derivatization agent.

All the CECs were analyzed by GC-MS/MS. Methylation of the acidic carboxyl group and the hydroxyls group of BPA for plant tissue was performed in a programmed temperature vaporizing (PTV) injector of the gas chromatograph by adding 10 µL TMSH to a 50 µL sample aliquot before injection. A volume of 5 µL was injected into a Bruker 450-GC gas chromatograph coupled to a Bruker 320-MS triple stage quadrupole mass spectrometer (Bruker Daltonics, Billerica, MA) fitted with a 20 m × 0.18 mm ID, 0.18 µm film thickness Sapiens X5-MS capillary column coated with 5 % diphenyl 95 % dimethyl polysiloxane from Teknokroma (Sant Cugat del Vallès, Spain). The PTV was set at 60 ºC for 0.5 min and rapidly heated to 300 ºC at 200 ºC min^{-1}, and hold for 7 min. Then the injector was cooled to initial 60 ºC at 200 ºC min^{-1}. The oven temperature was held at 60 ºC for 3.5 min and then the temperature was programmed at 30 ºC min^{-1} to a 150 ºC and finally at 8 ºC min^{-1} to 320 ºC, holding the final temperature for 6 minutes. Gas flow rate was set at 0.6 mL min^{-1}. Ion source temperature and the transfer line both were held at 250ºC. A solvent delay of 8 minutes was applied. Argon gas was used for CID at a pressure of 1.8 mTorr and the optimum collision energy (CE) was selected for each transition.

Qualitative and quantitative analysis was performed based on retention time and selected reaction monitoring (SRM) mode of two product ions, and the ratio between the product ions (Table S5). The limit of detection (LOD) and the limit of quantitation (LOQ) for plant tissue were defined as the mean background noise in a blank triplicate plus three and ten times,

respectively, the standard deviation of the background noise from three blanks. LODs and LOQs were compound dependent and ranged from 0.3 to 4.5 ng g^{-1} dry weight (Table S6). The recoveries of the surrogates added can be seen in Table S7.

Chlorophyll determination

Chlorophyll concentration was calculated with the following equations:

Chlorophyll A = $(12.70*Abs^{664.5}) - (2.79*Abs^{647})$ (Eq. S1)

Chlorophyll B = $(20.70*Abs^{647}) - (4.62*Abs^{664.5})$ (Eq. S2)

Total chlorophylls = $(17.90*Abs^{647}) - (8.08*Abs^{664.5})$ (Eq. S3)

where Abs is the absorbance at the specified wavelength (647 and 664.5 nm), and dividing it for the foliar surface.

Finally, soil pH was measured at a soil-water ratio of 1:5 $CaCl_2$ 0.01 M with a Crison GLP 22 pH meter equipped with a gel filled pH electrode IntelliCAL™ PHC101 (Hach Lange, CO, USA).

Data arrangement, compression and MCR-ALS analysis

Due to huge size of data sets collected in GC×GC-MS data sets of 20 lettuce samples, a data segmentation strategy was used. In this regard, GC×GC-TOFMS data for 20 samples segmented into four parts (A-D) by visual inspection of the chromatograms. Figure S3 shows these chromatographic segments as an example on GC×GC-TIC of one of the control sample.

Wavelet decomposition and compression is applied on every column (m/z) of $\mathbf{X}_{aug}$ independently. Compression reduces the size of data 2^n times, which, n is the compression level[1,2]. The compressed matrix ($\mathbf{X}_{compr}$) contains the same information as $\mathbf{X}_{aug}$, but needs much lower computer storage. For the datasets under study in this paper, level-3 wavelet

compression was sufficient without loss of relevant information in the elution time direction, and spectral domain remained unchanged.

Before starting MCR-ALS analysis, some prior knowledge is required. One of the main difficulties in MCR analysis is determination of the number of chemical components exist in data matrix. In this work, the size of singular values and changes in lack of fit (LOF) of MCR-ALS solutions by adding or removing components into the model were used as criteria to estimate the number of significant components.

To start ALS optimization, simple-to-use interactive self-modeling mixture analysis (SIMPLISMA)[3] was used to estimate the initial values of mass spectral profiles. In addition, proper constraints involving non-negativity (concentration and spectral modes), unimodality (concentration mode), spectra normalization (to unit length) and component correspondence (if applicable) were applied during ALS optimization to obtain reliable results with minimum rotational ambiguity[4]. It is important to note that the unimodality constraint was only applied to chemical components and not to baseline/background contributions. Component correspondence constraint was applied in the case of simultaneous analysis of several data matrices. This constraint fixes the sequence and presence, absence and correspondence of each component in the different data submatrices (modulations) of the super-augmented data matrix. This presence/absence information is coded in a binary format (1 or 0) and introduced into the MCR-ALS algorithm. As a consequence, when a component does not exist in a particular submatrix of super-augmented data matrix, the elements in the related data matrix are set to zero. This constraint can reduce significantly rotational ambiguities and gives more component selectivity. In addition, this constraint in fact matches reasonably well with the nature of GC×GC-TOFMS data where one component could exist in some modulations and be absent in others. The $\mathbf{C}_{aug}$ contains second dimension elution profiles for all N components in K modulations in L samples. To get first dimension elution profiles of every component in

each analyzed sample, every column in $\mathbf{C}_{aug}$ should be appropriately refolded to give a matrix for each analyzed sample. The column sum of this refolded data matrix gives an estimation of the corresponding first dimension elution profile. Therefore, for every sample, a matrix of first dimension elution profiles is obtained. Resolved second dimension elution profiles can be used for quantitative purposes.

The four column-wise super-augmented data matrix for four segments (A-D) in 20 samples were then analyzed using MCR-ALS. The MCR bilinear model for a data matrix such as the one taken from a modulation of the first column in GC×GC-TOFMS is based on the description of the variation of the measurements as a linear mixture of the contributions of their pure components[5]. The MCR bilinear model can be straightforwardly extended to higher order data, i.e., to the GC×GC-TOFMS data sets obtained in the analysis of different samples and arranged in a super-augmented data matrix. In linear algebra notation, the general MCR bilinear model applied to a super-augmented GC×GC-TOFMS data set obtained in the simultaneous analysis of multiple samples is as follow:

$$\mathbf{X}_{aug} = \mathbf{C}_{aug}\mathbf{S}^{T} + \mathbf{E}_{aug} \qquad \text{(Eq. S4)}$$

where $\mathbf{X}_{\mathbf{aug}}$(KLI,J) is column-wise super-augmented GC×GC-TOFMS data matrix with K data modulations taken from the first column with I rows (second column elution time points) and J columns (m/z values) for L samples. The $\mathbf{C}_{\mathbf{aug}}$ (KLI×N) is the super-augmented matrix containing resolved second dimension elution profiles for the different modulations. Note that the profiles of the same component in the different modulations may be different, both in shape and in peak position. The $\mathbf{S}^{T}$(N×J) is the matrix of common (invariant) pure mass spectra, and $\mathbf{E}_{\mathbf{aug}}$ (KLI×J) is the residual matrix with the data variance unexplained by the bilinear model $\mathbf{C}_{\mathbf{aug}}\mathbf{S}^{T}$. In addition, N is the number of chemical components considered in the factor matrices. MCR-ALS solves Eq. S4 for $\mathbf{C}$ and $\mathbf{S}^{T}$, using an iterative algorithm based on two constrained linear least-squares steps[6,7]. The values of coefficient of

determination (R^2) and LOF were used for evaluation of MCR-ALS model and they can be defined as follows:

$$R^2(\%) = \sqrt{\frac{\sum_{i=1}^{I}\sum_{j=1}^{J}\sum_{k=1}^{K}(\hat{x}_{aug,i,j,k})^2}{\sum_{i=1}^{I}\sum_{j=1}^{J}\sum_{k=1}^{K}x_{aug,i,j,k}^2}} \times 100$$

(Eq. S5)

$$LOF(\%) = \sqrt{\frac{\sum_{i=1}^{I}\sum_{j=1}^{J}\sum_{k=1}^{K}(x_{aug,i,j,k} - \hat{x}_{aug,i,j,k})^2}{\sum_{i=1}^{I}\sum_{j=1}^{J}\sum_{k=1}^{K}x_{aug,i,j,k}^2}} \times 100 \quad \text{(Eq. S6)}$$

where $x_{aug.i.j.k}$ is the element of the original data matrix and $\hat{x}_{aug.i.j.k}$ is the recovered value using MCR-ALS method.

Metabolite detection and NIST identification

MCR-ALS resolved profiles ($\mathbf{S}^T$) were assigned to metabolites and identified by comparing the mass fragmentation patterns associated to the MCR-ALS resolved mass spectra profiles using the standard mass spectral database of the National Institute of Standards and Technology (NIST) and GOLM. For each mass spectrum, 100 hits were retrieved by the NIST Mass Spectral Search 2.2 software distributed with the NIST 2014 library. A reverse match factor (RMF) based on the correlation coefficient between the MCR-AS resolved and experimental mass spectra reported by NIST software was used for selection of the best identified compound for MCR-ALS resolved mass spectra. This match factor is reported between 0 (no match) and 1000 (perfect match). As a general guide, a value of 900 or greater was considered to be a very good matching; between 800 and 900, a good match; between 700 and 800, a fair match; and less than 600 a poor or very poor match.

PLS modeling

Root mean squares error in leave-one-out cross-validation (RMSECV-LOO) has been used for choosing the significant number of latent variables (LV) in PLS model[8]. Root mean squares error in prediction (RMSEP) and relative error in prediction (REP) were the quantitative measures of prediction validity. The knowledge of the presence of some individual samples and/or variables which are mainly influential for a given model is very important. For a straightforward interpretation of the PLS2 model, variable importance in projection (VIP)[9] was used instead of commonly used weights and regression coefficients vectors. This method is based on the obtained PLS2 loading weights for variables. The VIP scores for each variable (j) is equal to its accumulated weights from all the selected LVs. This value is calculated as:

$$VIP_j = \sqrt{\frac{J.\sum_{f=1}^{F} w_{jf}^2 .SS_f}{SS_y.F}} \qquad \text{(Eq. S7)}$$

where SS_f and SSy are the sum of squares of the explained variance for the fth LV and total sum of squares of response matrix, respectively. Also, w_{jf} is the weight of the variable (j) on the fth LV and J and F are total number of the variables and LVs, respectively. Since the average of squared VIP scores equals 1, "greater than one rule" is generally used as a criterion for variable selection. Therefore, **X**-variables that have a VIP larger than one are important. With the aid of VIP scores, it is possible to determine the most influential variables among a huge number of variables in **X**-block.

The VIP gives information about how the variables combine to form the quantitative relation between **X** and **Y**, thus providing an interpretation of the scores. Hence, these VIP scores are essential for the understanding of which **X**-variables are important (numerically large *VIP* values), and which **X**-variables that provide the same information (similar profiles of *VIP*

values). A large VIP value in a chromatographic region indicates that the compounds eluted in that retention time region have a large impact on the prediction model, while, on the contrary, a low value indicates less influential components.

Example the MCR-ALS analysis (control and exposed samples)

Figures S2a and S2b show the resolved elution profiles in first and second chromatographic dimensions, respectively. Also, Fig S2c depicts the resolved mass spectra for 20 components. In Fig S2d, second column elution profiles for one of the resolved metabolite in 20 samples are demonstrated. As it can be seen, this metabolite has a very low concentration in control sample. However, its concentration increases by increasing the concentration of contaminant exposed to the lettuce samples. Using the MCR-ALS resolved mass spectrum for this metabolite and by comparing with NIST MS database and GOLM Metabolome database this metabolite was identified as L-5-Oxoproline (2TMS) (RMF=949). Other metabolites were also identified by comparing their MCR-ALS resolved mass spectra with NIST and GOLM databases

TABLES

Table S1. Percent variance captured by PLS model.

	**X**-block		**Y**-block	
LV	Individual	Total	Individual	Total
1	23.09	23.09	64.35	64.35
2	23.90	46.99	10.25	74.61
3	31.84	78.83	1.41	76.02
4	6.63	85.46	3.78	79.80

Table S2. Number of finally resolved peaks using MCR-ALS model related to lettuce metabolome and their corresponding information including chemical name, derivatization order, empirical formula and RMF

No	Metabolite	Derivatization	Formula	RMF
**1**	L-5-Oxoproline	2TMS	C11H23NO3Si2	949
**2**	Succinic acid	2TMS	C10H22O4Si2	935
**3**	Glyceric acid	3TMS	C12H30O4Si3	965
**4**	Phosphoric acid	3TMS	C9H27O4PSi3	858
**5**	Fumaric acid	2TMS	C10H20O4Si2	915
**6**	Glycerol	3TMS	C12H32O3Si3	939
**7**	Malic acid	2TMS	C10H22O5Si2	880
**8**	meso-Erythritol	4TMS	C16H42O4Si4	886
**9**	2,3-Butanediol	2TMS	C10H26O2Si2	817
**10**	Threonic acid	4TMS	C16H40O5Si4	880
**11**	Methylmalonic acid	2TMS	C10H22O4Si2	862
**12**	L-Serine	2TMS	C9H23NO3Si2	774
**13**	3-Methyl-2-oxovaleric acid	1TMS	C9H18O3Si	796
**14**	bis-1,2-acetin ether	2TMS	C11H26O4Si2	672
**15**	Tartaric acid	4TMS	C16H38O6Si4	887
**16**	Ribofuranose	4TMS	C17H42O5Si4	874
**17**	Citric acid	4TMS	C18H40O7Si4	836
**18**	Xylose	4TMS	C20H52O5Si5	749
**19**	Quinic acid	5TMS	C22H52O6Si5	791
**20**	Ribitol	5TMS	C20H52O5Si5	795
**21**	Galactose	4TMS	C19H47NO5Si4	849
**22**	Tagatose	5TMS	C22H55NO6Si5	745
**23**	Sorbose	5TMS	C22H55NO6Si5	753
**24**	Arabinose	4TMS	C19H47NO5Si4	767
**25**	Methyl-4-O-methyl-α-D-glucopyranoside	3TMS	C17H40O6Si3	823
**26**	Ribose	4TMS	C17H42O5Si4	823
**27**	Lyxose	4TMS	C18H45NO5Si4	748
**28**	Glucose	5TMS	C22H55NO6Si5	920
**29**	Inositol isomer 4	6TMS	C24H60O6Si6	972
**30**	2-O-Glycerol-α-d-galactopyranoside	6TMS	C27H66O8Si6	857
**31**	Gluconic acid	6TMS	C24H60O7Si6	907
**32**	Glucopyranose	5TMS	C21H52O6Si5	870
**33**	D-Allose	5TMS	C22H55NO6Si5	843
**34**	Inositol isomer 1	6TMS	C24H60O6Si6	822
**35**	Ribonic acid	5TMS	C20H50O6Si5	800
**36**	Inositol isomer 2	6TMS	C24H60O6Si6	841
**37**	Inositol isomer 3	6TMS	C24H60O6Si6	889
**38**	Galactitol	6TMS	C24H62O6Si6	742

**39**	Mannonic acid, γ-lactone	4TMS	C18H42O6Si4	762
**40**	Galactonic acid	6TMS	C18H42O6Si4	801
**41**	(S,S,R,R,S)- methyl 6-deoxy-β-L-Galactopyranoside	3TMS	C16H38O5Si3	802
**42**	Mannose	5TMS	C22H55NO6Si5	817
**43**	Allo-Inositol	6TMS	C24H60O6Si6	842
**44**	beta-D-Galactopyranoside-(1,2)-glycerol	6TMS	C27H66O8Si6	859
**45**	Trehalose	8TMS	C36H86O11Si8	839
**46**	Adenosine	3TMS	C19H37N5O4Si3	731
**47**	Ethyl α-D-glucopyranoside	4TMS	C20H48O6Si4	771
**48**	Tagatofuranose	5TMS	C21H52O6Si5	771
**49**	Melibiose	8TMS	C36H86O11Si8	746
**50**	Sucrose	8TMS	C35H84O11Si8	842

Table S3. Pathway Search Results from KEGG (1)

ath0110 Metabolic pathways - (22)	
C00009	Orthophosphate
C00031	D-Glucose
C00042	Succinate
C00065	L-Serine
C00089	Sucrose
C00116	Glycerol
C00122	Fumarate
C00124	D-Galactose
C00137	myo-Inositol
C00149	(S)-Malate
C00158	Citrate
C00159	D-Mannose
C00181	D-Xylose
C00212	Adenosine
C00257	D-Gluconic acid
C00258	D-Glycerate
C00259	L-Arabinose
C00474	Ribitol
C00671	(S)-3-Methyl-2-oxopentanoic acid
C00880	D-Galactonate
C01083	Trehalose
C01697	Galactitol
ath02010 ABC transporters - (14)	
C00009	Orthophosphate

C00031	D-Glucose
C00065	L-Serine
C00089	Sucrose
C00116	Glycerol
C00121	D-Ribose
C00137	myo-Inositol
C00159	D-Mannose
C00181	D-Xylose
C00259	L-Arabinose
C00503	Erythritol
C01083	Trehalose
C01487	D-Allose
C05402	Melibiose
ath01110 Biosynthesis of secondary metabolites - (10)	
C00031	D-Glucose
C00042	Succinate
C00065	L-Serine
C00122	Fumarate
C00149	(S)-Malate
C00158	Citrate
C00257	D-Gluconic acid
C00258	D-Glycerate
C00671	(S)-3-Methyl-2-oxopentanoic acid
C01083	Trehalose
ath00052 Galactose metabolism - (10)	
C00031	D-Glucose
C00089	Sucrose
C00116	Glycerol
C00124	D-Galactose
C00137	myo-Inositol
C00159	D-Mannose
C00795	D-Tagatose
C00880	D-Galactonate
C01697	Galactitol
C05402	Melibiose
ath01200 Carbon metabolism - (7)	
C00042	Succinate
C00065	L-Serine
C00122	Fumarate
C00149	(S)-Malate
C00158	Citrate
C00257	D-Gluconic acid

C00258	D-Glycerate
ath00630 Glyoxylate and dicarboxylate metabolism - (6)	
C00042	Succinate
C00065	L-Serine
C00149	(S)-Malate
C00158	Citrate
C00258	D-Glycerate
C00552 meso-Tartaric acid	
ath00040 Pentose and glucuronate interconversions - (5)	
C00116	Glycerol
C00181	D-Xylose
C00259	L-Arabinose
C00474	Ribitol
C00476	D-Lyxose
ath00030 Pentose phosphate pathway - (4)	
C00031	D-Glucose
C00121	D-Ribose
C00257	D-Gluconic acid
C00258	D-Glycerate
ath00520 Amino sugar and nucleotide sugar metabolism - (4)	
C00031	D-Glucose
C00159	D-Mannose
C00181	D-Xylose
C00259	L-Arabinose
ath00020 Citrate cycle (TCA cycle) - (4)	
C00042	Succinate
C00122	Fumarate
C00149	(S)-Malate
C00158	Citrate
ath01230 Biosynthesis of amino acids - (3)	
C00065	L-Serine
C00158	Citrate
C00671	(S)-3-Methyl-2-oxopentanoic acid
ath00620 Pyruvate metabolism - (3)	
C00042	Succinate
C00122	Fumarate
C00149	(S)-Malate
ath00500 Starch and sucrose metabolism - (3)	
C00031	D-Glucose
C00089	Sucrose
C01083	Trehalose
ath00250 Alanine, aspartate and glutamate metabolism - (3)	

C00042	Succinate
C00122	Fumarate
C00158	Citrate
ath00053 Ascorbate and aldarate metabolism - (3)	
C00137	myo-Inositol
C00259	L-Arabinose
C01620	Threonate
ath00190 Oxidative phosphorylation - (3)	
C00009	Orthophosphate
C00042	Succinate
C00122	Fumarate
ath00650 Butanoate metabolism - (3)	
C00042	Succinate
C00122	Fumarate
C03044	(R,R)-Butane-2,3-diol
ath00051 Fructose and mannose metabolism - (3)	
C00159	D-Mannose
C00247	L-Sorbose
C01487	D-Allose
ath00350 Tyrosine metabolism - (2)	
C00042	Succinate
C00122	Fumarate
ath00562 Inositol phosphate metabolism - (2)	
C00137	myo-Inositol
C19891	1D-chiro-Inositol
ath00260 Glycine, serine and threonine metabolism - (2)	
C00065	L-Serine
C00258	D-Glycerate
ath00360 Phenylalanine metabolism - (2)	
C00042	Succinate
C00122	Fumarate
ath00760 Nicotinate and nicotinamide metabolism - (2)	
C00042	Succinate
C00122	Fumarate
ath00280 Valine, leucine and isoleucine degradation - (2)	
C00671	(S)-3-Methyl-2-oxopentanoic acid
C02170	Methylmalonate
ath01210 2-Oxocarboxylic acid metabolism - (2)	
C00158	Citrate
C00671	(S)-3-Methyl-2-oxopentanoic acid
ath00640 Propanoate metabolism - (2)	
C00042	Succinate

C02170	Methylmalonate
ath00561 Glycerolipid metabolism - (2)	
C00116	Glycerol
C00258	D-Glycerate
ath00920 Sulfur metabolism - (2)	
C00042	Succinate
C00065	L-Serine

1) Pathways with less than two metabolites detected are not included. Pathway dataset from *Arabidopsis thaliana.*

Table S4. Physical-chemical properties of the CECs. All values were obtained using ACD Advanced Chemistry Development (2010), ACD/i-lab 2.0. Toronto, 2010.

**CEC**	**Molar mass (g mol^{-1})**	**p*K*a**	**Speciation (z)**	**Neutral log K_{OW}**	**Solubility (mg mL^{-1})**
Benzophenone (BZP)	182.22	NA	0	2.98	0.17
Bisphenol A (BPA)	250.27	7.7<br>8.5	0/-1/-2	2.01	0.13
Butylated hydroxytoluene (BHT)	220.35	12.1	0/-1	5.06	0.0046
Caffeine (CAF)	194.19	NA	0	0.11	21.5
Carbamazepine (CBZ)	236.27	NA	0	2.23	0.084
Methyl paraben (MePB)	152.15	8.5	0/-1	1.97	1.3
5-Methyl-1H-benzotriazole (MeBT)	133.15	1.6<br>8.5	+1/0/-1	1.57	3.7
4-Octylphenol (OPL)	206.32	10.0	0/-1	5.64	0.0026
Phenazone (PZE)	188.23	1.8	+1/0	0.85	75.8
Triclosan (TCS)	258.40	8.8	0/-1	5.21	0.0046
Tris(2-chlorethyl) phosphate (TCP)	285.49	NA	0	1.72	5.0

NA: Not applicable; z is charge number (valence) and K_{OW} (L/L) is the partition coefficient octanol to water for the neutral molecule.

Table S5. Monitoring ions in GC-MS/MS

Segment	Compound	RT (min)	Precursor ion (m/z)	Product ion (m/z)	Collision energy (eV)
1	MePB	9.06	166*	135	13
			135	77	18
1	BHA	9.41	194*	179	14
			179	149	14
2	EPB	9.83	180*	152	11
			135	77	18
3	BHT	10.40	220*	205	17
			205	177	12
3	MeBT	10.36	147*	118	14
			118	77	17
4	OPL	10.91	149*	121	15
			135	77	18
5	BZP	12.04	182*	105	17
			105	77	14
5	XTTri	12.23	161*	132	16
			132	91	18
6	TCP	12.65	249*	125	15
			249	99	30
7	CAF	14.65	194*	109	14
			194	55	20
7	CAF-$^{13}C_3$	14.61	197*	110	12
			110	82	17
7	PZE	15.21	188*	159	11
			188	96	15
8	CBZ	16.44	193*	191	23
			193	167	18
8	CBZ-$^{13}C_6$	16.40	199*	173	25
			199	197	20
8	TPhA	16.78	245*	167	30
			245	141	21
9	BPA	17.11	241*	133	15
			241	211	17
9	BPA-d6	17.09	270*	252	14
			252	139	20
10	TCS	17.86	302*	252	19
			302	189	37

* Transition used for quantification

Table S6. Limits of detection (LOD) and quantification (LOQ) of the selected CECs in the lettuce leaves.

Compound	LOD ($ng\ g^{-1}$ dw)	LOQ ($ng\ g^{-1}$ dw)
BHT	0.9	1.1
BPA	0.7	0.9
BZP	1.8	2.3
CAF	1.7	1.9
CBZ	1.0	1.5
MeBT	1.1	1.3
MePB	0.3	0.4
OPL	2.9	4.5
PZE	2.4	3.1
TCP	0.7	1.0
TCS	1.4	1.7

Table S7. Average recoveries of the surrogates added in each compartment and the SD of all the samples (N = 20).

Compound	Recovery (%)
BHA	67 ± 4
BPA-$^{13}C_6$	78 ± 5
CAF-$^{13}C_3$	52 ± 3
CBZ-$^{13}C_6$	69 ± 7
EPB	73 ± 5
XTTri	65 ± 4

Table S8. Log fold ratios of control in front of the four CECs exposure concentrations 0.05, 0.5, 5 and 50 µg L^{-1}.

Metabolite	Control vs 0.05 µg L^{-1}	Control vs 0.5 µg L^{-1}	Control vs 5 µg L^{-1}	Control vs 50 µg L^{-1}
L-5-Oxoproline	0.61	0.82	0.96	0.97
L-Serine	0.03	0.14	0.19	0.29
3-Methyl-2-oxovaleric acid	-0.19	-0.20	-0.19	-0.11
Adenosine	0.14	0.03	0.08	0.17
Phosphoric acid	-0.16	-0.29	-0.13	-0.11
γ-lactoneMannonic acid	-0.10	-0.11	-0.07	0.00
Citric acid	0.28	0.21	0.15	0.23
Fumaric acid	-0.21	-0.08	-0.08	0.02
Galactonic acid	0.02	0.25	0.31	0.31
Gluconic acid	0.25	0.28	0.32	0.37
Glyceric acid	-0.15	-0.16	-0.20	-0.10

Malic acid	-0.50	-0.51	-0.49	-0.40
Methylmalonic acid	-0.09	-0.07	-0.08	0.02
Quinic acid	0.01	-0.08	-0.07	-0.04
Ribonic acid	-0.02	0.22	0.30	0.29
Succinic acid	0.07	0.10	0.05	0.19
Tartaric acid	0.17	0.00	0.07	0.12
Threonic acid	-0.14	-0.14	-0.14	-0.08
2,3-Butanediol	-0.38	-0.32	-0.32	-0.22
bis-1,2-acetin ether	-0.13	-0.08	-0.07	0.02
Allo-Inositol	-0.12	0.11	0.15	0.15
beta-D-Galactopyranoside-(1,2)-glycerol	0.23	-0.06	-0.12	-0.16
Galactitol	0.07	0.11	0.24	0.30
Glycerol	-0.12	-0.11	-0.12	-0.01
Inositol isomer 1	-0.10	0.19	0.24	0.23
Inositol isomer 2	-0.07	0.26	0.30	0.31
Inositol isomer 3	-0.18	0.09	0.13	0.13
meso-Erythritol	0.11	0.11	0.10	0.20
Inositol isomer 4	-0.02	0.03	0.06	0.07
Ribitol	0.15	0.09	0.11	0.06
(S,S,R,R,S)-methyl 6-deoxy-β-L-Galactopyranoside	-0.03	0.01	0.10	0.15
2-O-Glycerol-α-d-galactopyranoside	-0.11	-0.24	-0.21	-0.13
Allose	-0.47	-0.43	-0.30	-0.31
Arabinose	0.15	0.10	0.09	0.12
Ethyl α-D-glucopyranoside	0.26	0.01	-0.04	0.04
Galactose	0.02	-0.17	-0.14	-0.07
Glucopyranose	-0.05	0.22	0.26	0.35
Glucose	-0.68	-0.61	-0.39	-0.48
Lyxose	0.19	0.06	0.09	0.10
Mannose	-0.13	0.13	0.22	0.23
Melibiose	0.27	0.06	-0.01	0.00
Methyl-4-O-methyl-α-D-glucopyranoside	0.05	-0.14	-0.07	-0.05
Ribofuranose	0.13	-0.12	-0.09	-0.09
Ribose	0.09	-0.12	-0.08	-0.06
Sorbose	-0.20	-0.35	-0.29	-0.29
Sucrose; alpha-D-Glc-(1,2)-beta-D-Fru]	0.28	0.14	0.13	-0.12
Tagatofuranose	0.24	-0.15	-0.20	-0.27
Tagatose	0.20	0.08	0.08	0.07
Trehalose	0.42	-0.12	-0.11	-0.27
Xylose	-0.04	-0.21	-0.13	-0.13

FIGURES

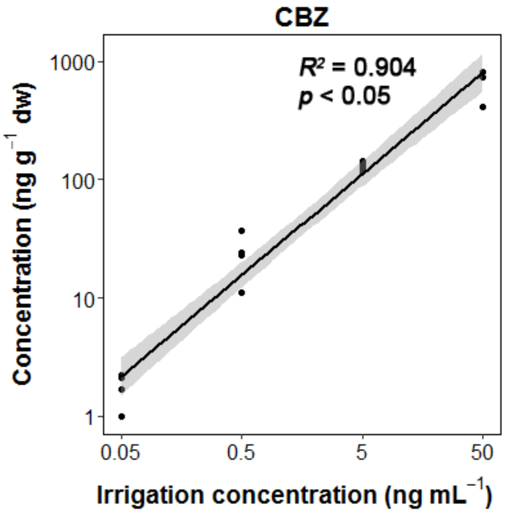

Figure S1. Linear correlation between the leaf concentration (ng g^{-1} dw) and the irrigation concentration (ng mL^{-1}) of CBZ. .

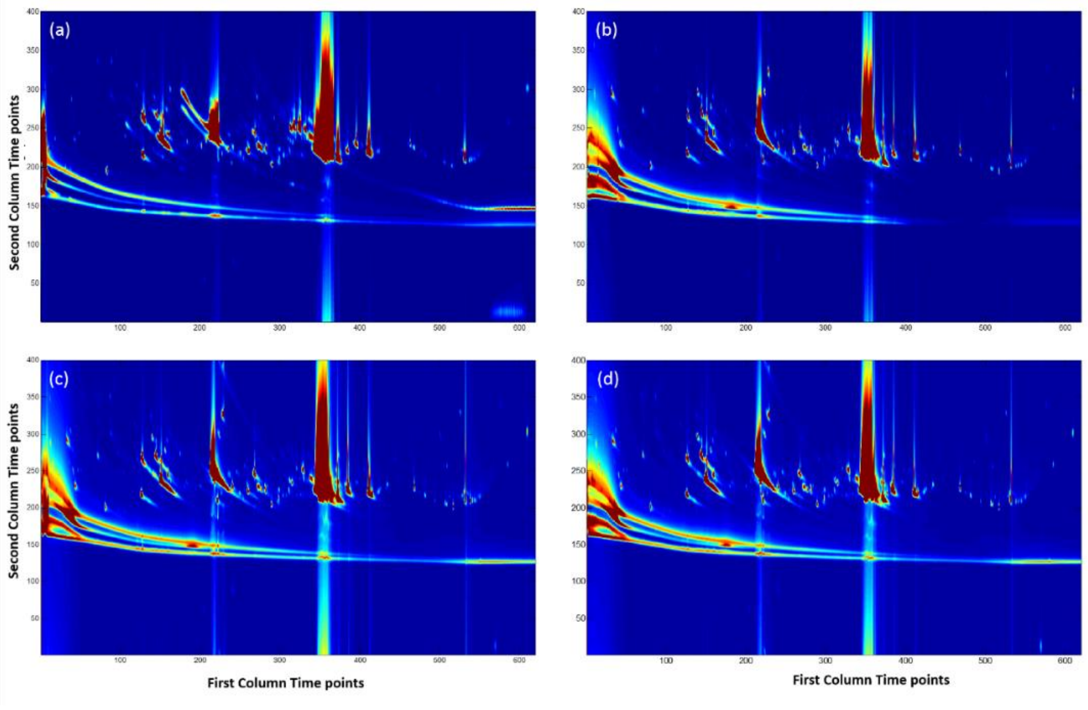

Figure S2. Contour plots of lettuce extracts analyzed by GC×GC–TOFMS. Lettuces exposed at (a) 0 µg L^{-1}, (b) 0.05 µg L^{-1}, (c) 0.5 µg L^{-1}, and (d) 50 µg L^{-1} of 11 CECs.

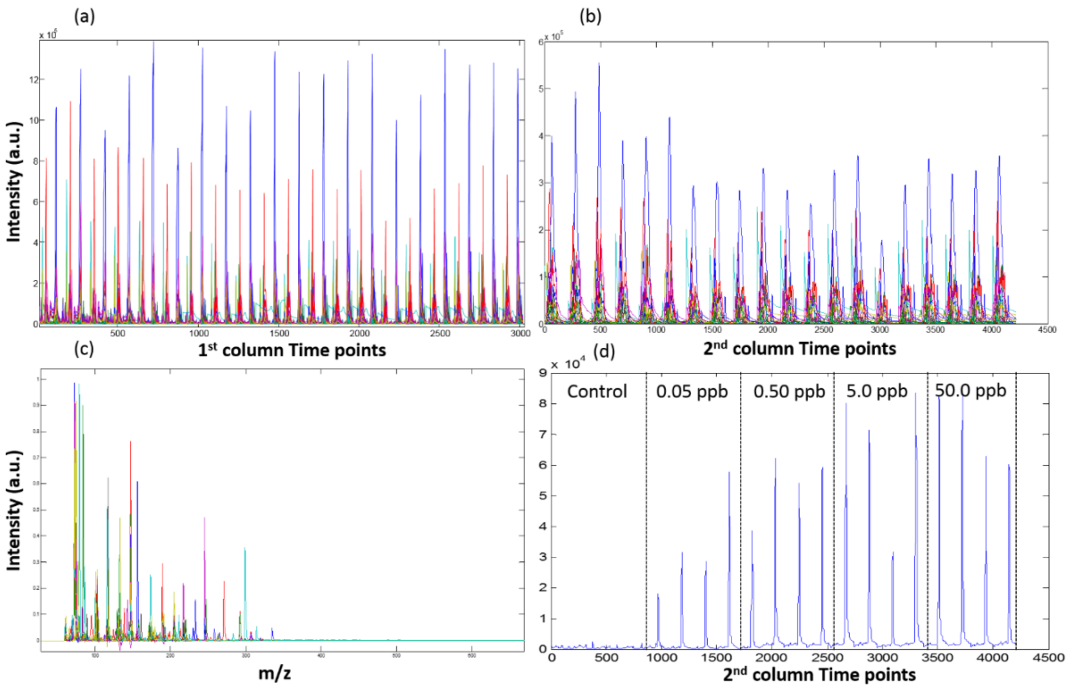

Figure S3. MCR-ALS analysis of segment 1 of 4 segments in 20 samples (control and exposed samples). The number of components was 20 in this case which confirmed using singular value decomposition (SVD). The value of lack of fit (LOF) and R^2 for the developed model were respectively 4.6% and 99.8%, which were acceptable according to the noise level of data.

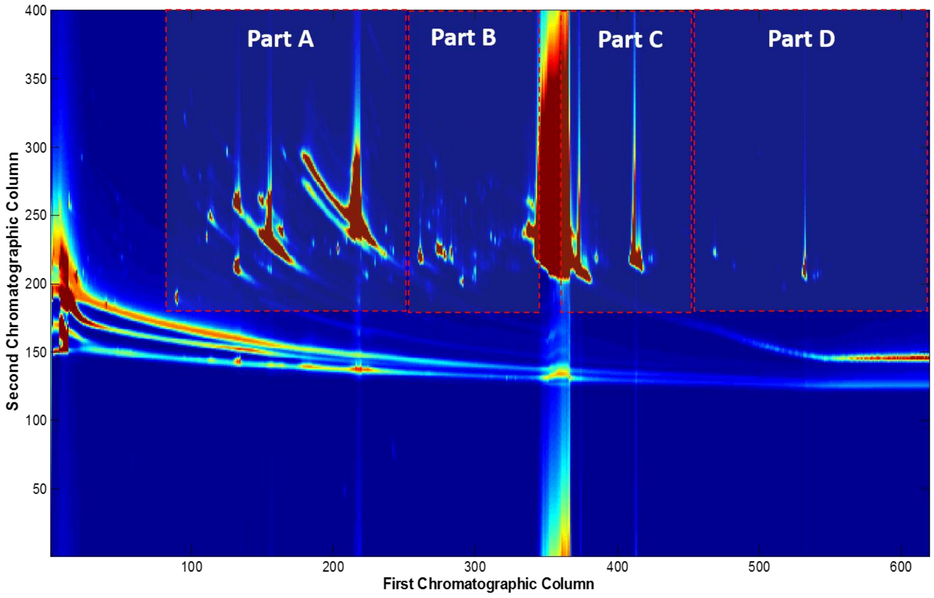

Figure S4. Total ion chromatogram (TIC) of GC×GC-MS data of one of the control samples. Four different chromatographic segments are shown in this figure.

REFERENCES

1 Walczak, B. & Massart, D. L. Wavelets - something for analytical chemistry? *TrAC, Trends Anal. Chem.* **16**, 451-462, (1997).

2 Shao, X., Cai, W. & Pan, Z. Wavelet transform and its applications in high performance liquid chromatography (HPLC) analysis. *Chemom. Intell. Lab. Syst.* **45**, 249-256, (1999).

3 Windig, W. & Guilment, J. Interactive self-modeling mixture analysis. *Anal. Chem.* **63**, 1425-1432, (1991).

4 Tauler, R. Calculation of maximum and minimum band boundaries of feasible solutions for species profiles obtained by multivariate curve resolution. *J. Chemom.* **15**, 627-646, (2001).

5 Parastar, H. & Tauler, R. Multivariate curve resolution of hyphenated and multidimensional chromatographic measurements: a new insight to address current chromatographic challenges. *Anal. Chem.* **86**, 286-297, (2014).

6 Tauler, R., Smilde, A. & Kowalski, B. Selectivity, local rank, 3-way data analysis and ambiguity in multivariate curve resolution. *J. Chemom.* **9**, 31-58, (1995).

7 Tauler, R. Multivariate curve resolution applied to second order data. *Chemom. Intell. Lab. Syst.* **30**, 133-146, (1995).

8 Wold, S., Sjöström, M. & Eriksson, L. PLS-regression: A basic tool of chemometrics. *Chemom. Intell. Lab. Syst.* **58**, 109-130, (2001).

9 Chong, I. G. & Jun, C. H. Performance of some variable selection methods when multicollinearity is present. *Chemom. Intell. Lab. Syst.* **78**, 103-112, (2005).
